# What 'outliers' tell us about missed opportunities for tuberculosis control: a cross-sectional study of patients in Mumbai, India

**DOI:** 10.1186/1471-2458-10-263

**Published:** 2010-05-20

**Authors:** Anagha Pradhan, Karina Kielmann, Himanshu Gupte, Arun Bamne, John DH Porter, Sheela Rangan

**Affiliations:** 1 Centre for Health Research and Development, Maharashtra Association of Anthropological Sciences, 64-5 Anand Park, Pune - 411007, India; 2 Health Policy Unit, London School of Hygiene and Tropical Medicine, Keppel Street, London WC1E 7HT, UK; 3 Inter Aide Development India, 24E Sarojini Road, Mumbai - 400054, India; 4 Mumbai District TB Control Society and Municipal Corporation of Greater Mumbai, Dr. E. Moses Road, Worli, Mumbai - 400018, India

## Abstract

**Background:**

India's Revised National Tuberculosis Control Programme (RNTCP) is deemed highly successful in terms of detection and cure rates. However, some patients experience delays in accessing diagnosis and treatment. Patients falling between the 96^th ^and 100^th ^percentiles for these access indicators are often ignored as atypical 'outliers' when assessing programme performance. They may, however, provide clues to understanding why some patients never reach the programme. This paper examines the underlying vulnerabilities of patients with extreme values for delays in accessing the RNTCP in Mumbai city, India.

**Methods:**

We conducted a cross-sectional study with 266 new sputum positive patients registered with the RNTCP in Mumbai. Patients were classified as 'outliers' if patient, provider and system delays were beyond the 95^th ^percentile for the respective variable. Case profiles of 'outliers' for patient, provider and system delays were examined and compared with the rest of the sample to identify key factors responsible for delays.

**Results:**

Forty-two patients were 'outliers' on one or more of the delay variables. All 'outliers' had a significantly lower per capita income than the remaining sample. The lack of economic resources was compounded by social, structural and environmental vulnerabilities. Longer patient delays were related to patients' perception of symptoms as non-serious. Provider delays were incurred as a result of private providers' failure to respond to tuberculosis in a timely manner. Diagnostic and treatment delays were minimal, however, analysis of the 'outliers' revealed the importance of social support in enabling access to the programme.

**Conclusion:**

A proxy for those who fail to reach the programme, these case profiles highlight unique vulnerabilities that need innovative approaches by the RNTCP. The focus on 'outliers' provides a less resource- and time-intensive alternative to community-based studies for understanding the barriers to reaching public health programmes.

## Background

Mumbai's Revised National Tuberculosis Control Programme (RNTCP) is a shining illustration of the Indian national tuberculosis (TB) control programme, the world's largest and reportedly highly successful national TB control programme [1]. In 2008, the Mumbai programme enrolled more than 30,000 patients with a case detection rate of 85% and cure rate of 87% [2]. The RNTCP uses **DOTS**, the World Health Organisation's TB control strategy since 1995 (branded in 2005) that relies on a standardised regimen administered under direct observation of patients. For most patients, the time between onset of symptoms and contact with the first provider (patient delay) is very short, with a longer time lag between contact with the first provider and entry into the programme (provider delay). Once the patients enter the RNTCP, system delays (diagnostic delay and treatment delay) are minimal. For each of these four indicators of access to TB care, three to five percent of the patients experience delays that fall between the 96^th ^and 100^th ^percentile values for respective delay variables. For the purpose of this paper, they are referred to as 'outliers', representing the extreme values that lie beyond what is considered acceptable for delays in the TB care seeking process, based on the available literature on delays. They are often ignored as rare occurrences or atypical cases when assessing programme performance.

Far from being atypical, we suggest that these patients provide us with important clues about why some patients will never reach the programme. As indicated elsewhere, patients who do not get on to DOTS form a particularly vulnerable group with respect to TB control [3,4]. While successful programmes like the Mumbai RNTCP are designed and funded to address the health needs of the poor, economic disadvantage often conflates with other vulnerabilities that are less easy to recognize and leads to individuals being 'missed out' by disease control programmes [5].

Mumbai is the largest metropolis and financial capital of India and one of the most crowded cities in the world. Just over half of its inhabitants (54%) reside in slums; many are migrants drawn to Mumbai by job opportunities in the city's vast unorganized sector [6,7]. In the decade between 1991 and 2001, 54.8% of the population growth in Mumbai was due to in-migration from outside the state [8]. In these crowded urban environments, individuals' vulnerability to infectious diseases including TB is greatly increased [9-12]. TB is the third most common cause of mortality in Mumbai, accounting for almost one tenth of the total deaths reported in the city (Personal communication, Public Health Department of Municipal Corporation of Greater Mumbai).

While RNTCP services are provided through municipal (public) and accredited (private) microscopy centres and DOT (directly observed treatment) centres established within municipal health care facilities, private providers are the first point of contact for most TB patients following onset of symptoms [13]. Acknowledging the role of the private sector in TB care, the RNTCP in Mumbai has formally involved high numbers of private practitioners (PP) and non-governmental organizations (NGO) that serve as DOT providers [14]. Patients generally reach the free TB care services provided through the RNTCP only after exhausting their resources in the private sector.

This paper draws on a study that explored access of the urban poor to the RNTCP in Mumbai, India (MAAS-CHRD, Access to TB care through the RNTCP - Summary Report, unpublished). Against the background of a highly successful programme, close attention to the experiences of these patients can elucidate the nature of vulnerabilities that underlie delays and missed opportunities for timely access to effective treatment.

## Methods

### Study design and sampling

A cross-sectional study was carried out between May 2005 and February 2006. The metropolis of Mumbai is divided into 24 administrative units called wards with population of approximately 500,000 each. The sampling frame for the study component on which this paper is based was formed by new sputum positive (NSP) patients registered under the RNTCP at DOT centres in seven of the 14 slum dominant wards. The first quarter of 2005 was used as the reference period for determining the sample size. In the selected wards, the DOT centres were classified into 'low', 'medium', and 'high' based on their contribution to the ward-level case load. A stratified random sampling method was used to select DOT centres from each ward. All patients from each of the selected DOT centre who had completed between six and 16 doses were included in the study. The sample thus consisted of 266 NSP patients (15% of the quarterly case load). All NSP patients registered for treatment in the three weeks preceding the period of data collection were included in the sample. Patients less than 15 years of age, too ill to speak and who were admitted to the hospital were excluded from the study.

The study was approved by the Institutional Ethics Committee of the Maharashtra Association of Anthropological Sciences, Pune, India and the Institutional Review Board of the London School of Hygiene and Tropical Medicine, London, UK.

### Data collection

After obtaining informed consent, respondents were interviewed by trained investigators (eight of ten had post-graduate training in social sciences) using a semi-structured interview schedule with roughly two-thirds of the questions framed in an open-ended manner. The interview schedule elicited information on socio-economic background, help-seeking behaviour including when, why and what type of providers were consulted, duration of treatment with each provider, investigations advised, cost of treatment and reason for discontinuing treatment with each provider, experiences at the microscopy and DOT centres, social support received and opinions about direct observation of treatment. We did not, however, elicit the exact nature of the treatment received by the patients. The majority of interviews (243/266, 91%) were conducted at the DOT centre. Twenty-three patients (9%) were interviewed at locations other than the DOT centre, convenient to the respondent. Privacy was ensured, wherever possible, by interviewing respondents in a location where others could not overhear the interview. Respondents unable to speak due to breathlessness or drowsiness following consumption of medicines were contacted more than once to complete the interview. All respondents were interviewed in the intensive phase of DOTS. Respondent names were kept confidential and their details were not disclosed to the DOT providers and the programme managers.

### Data management and analysis

Quantitative data were coded and analysed using SPSS, Version 10 (SPSS, Chicago Inc, IL, USA). Twenty percent of all data entry forms were cross-checked for coding and data entry errors independently by two researchers. 'Outliers' were identified from the ranges on patient, provider, diagnostic and treatment delays. Patient delay was defined as the period between onset of symptoms and first contact with a formal health care provider. Provider delay was defined as the duration between the first contact with the first provider and first visit to the microscopy centre. Diagnostic delay was defined as the duration between the first visit to the microscopy centre and collection of the report. Treatment delay was defined as the duration between the diagnosis (receipt of reports of sputum examination for acid-fast bacilli (AFB) and the first dose of the DOTS.

Case profiles were reconstructed from respondents' open-ended narration of their treatment seeking pathways. Respondent names have been changed to maintain confidentiality; the names used in this paper are pseudonyms. Italicised patient quotations are verbatim translations of open-ended answers provided by respondents.

## Results

### Cases available for analysis

The valid number of cases available for analysis for each of the delays is presented in Table 1. For patient and provider delays, cases in which respondents were unable to provide details for all help seeking contacts were excluded. Thirty-eight respondents who underwent sputum examination while admitted to hospitals were excluded from analysis for diagnostic delay. For treatment delay, all except four respondents could provide data regarding duration between diagnosis and, receiving the first dose of DOTS. Respondents who were beyond the 95^th ^percentile value for the selected four indicators were listed and compared with the rest of the sample.

**Table 1:** Cases available for analysis

Indicator	Patient delay	Provider delay	Diagnostic delay	Treatment delay
Number of respondents asked the information	266	266	228	266

Number of respondents providing valid responses	255	197	216	262

Minimum - Maximum (days)	0 - 1825	1 - 754	0 - 91	0 - 47

Mean (days)	30	56	5	6

Median (days)	6	27	4	4

95^th ^percentile (days)	92.00	201.20	10.15	22.70

Number of respondents beyond 95^th ^percentile (% of valid responses)	11 (4%)	9 (5%)	10 (5%)	13 (5%)

### Patient profile

Median age for the respondents (n = 266) was 26 years (range 15 to 80 years, mean = 31 year); more than a third (99/266, 37%) had up to four years of schooling, less than half (108/266, 41%) were employed at the time of interview and came from households with a median monthly per capita income of 778.9 Indian rupees (US$ 17.3). Half of the respondents (134/266, 50%) had lived in Mumbai for ten or more years (Table 2).

**Table 2:** Profile of the study sample

Variable		Total
**Sex**		**n = 266**
	Female	110 (41%)
	Male	156 (59%)

**Age (years)**		**n = 266**
	Mean	31
	Median (Range)	26 (15 - 80)

**Marital status**		**n = 266**
	Unmarried	109 (41%)
	Married + Others	156 (59%)

**Employment status**		**n = 266**
	Employed	108 (41%)
	Unemployed + other	158 (59%)

**Monthly per capita income (Rs.)**		**n = 180**
	Mean	1019.85
	Median (Range)	778.89 (37.33 - 4000)

**Residence in Mumbai**		**n = 266**
	Upto 10 years	132(50%)
	More than 10 years	134 (50%)

**First provider**		**n = 266**
	Public sector	38 (14%)
	Private sector	219 (82%)
	Microscopy centre	9 (3%)

Note: Percentages are calculated on valid responses.

Forty-one respondents were 'outliers' for one of the delay variables; only one respondent was an 'outlier' for two of these variables. The 'outliers' for each of the four selected delay variables were compared with the rest of the sample to identify socio-economic variables that could help predict the 'outliers'.

### Patient delay

Two hundred and fifty-five of the 266 (96%) respondents interviewed for the study could provide valid information regarding patient delay and were included in the analysis related to patient delay (Table 1). Half of the respondents had consulted a first provider within six days of the onset of symptoms. Eleven percent (27/255) had sought a provider on the same day as the symptoms started, and more than three-quarters (210/255, 82%) had consulted a provider within 21 days of onset of symptoms (the cut-off point for suspicion of TB till revision of diagnostic guidelines in 2009). The 95^th ^percentile for the sample consisted of 11 respondents reporting a delay of more than 92 days. These respondents showed wide variation in most socio-demographic and economic characteristics such as age, education and employment status. However, the majority (10/11) had a per capita income of less than 1350 rupees per month (approximately 1 US$ per day) as compared with the rest of the sample (119/161, 74%) (Chi-square, p = 0.060). The difference between the outliers and the rest of the respondents was not statistically significant for any other socio-demographic and economic variables.

A patient's subjective evaluation of bodily signs as symptoms of an illness is the first crucial step - or barrier - to timely help-seeking. Six of the 11 respondents reported a gradual onset of symptoms which they did not "*pay attention*" to. Others, like 60-year old Baldev Singh and 45-year old Seetaram, failed to recognise the symptoms while under the influence of alcohol. Sixty-seven year old Babubhai, also on treatment for leprosy, attributed his cough with expectoration to "*many years of smoking lots of bidis *[hand-made local cigarettes]". Fifty-eight year old Shaikh Ahmed believed that his long term symptoms of expectoration, fever, and weight loss were the result of a "*chronic cold*". The experiences of these respondents highlight the role of patients' awareness and perceived gravity of their symptoms as important prompts in timely help-seeking.

### Provider delay

On average, respondents took close to four weeks (n = 197; median 27 days, range: 1 to 754 days) to reach the microscopy centre following contact with a first provider. The proportion of women (7/9) and patients with lower educational status (7/9) was higher among the 'outliers' as compared with the rest of the sample; women constituted 42% (78/188) and patients with lower educational status formed 32% (61/188) of the sample. Not only patients' help-seeking behaviour, but private sector practices contributed to the severe delays incurred by the nine respondents who fell beyond the 95^th ^percentile (201 days). With respect to consulting health providers, 'outliers' were similar to the rest of the sample: the average numbers of providers contacted and visits paid to these providers did not differ in the two groups. However, the data from the 'outliers' show longer gaps between provider consultations (Mann-Whitney Test, p = .000), suggesting that 'outliers' remained with the provider for a longer period than the rest of the sample. These longer periods of treatment resulted in costs roughly four times as much as those incurred by the rest of the sample: median costs for 'outliers' were Rs.1375 (US$ 30.6) versus only Rs.330 (US$ 7.3) for the rest of the sample (Mann-Whitney Test p = .014). These costs represent a larger financial burden on the households of 'outliers' who had lower per capita incomes than the rest of the individuals in the sample (Mann-Whitney Test p = .010) (Table 3).

**Table 3:** Help-seeking behaviour among 'outliers'

		Provider delay	
		**Upto 95**^**th **^**percentile**	**Beyond 95**^**th **^**percentile**
***Number of providers visited before Microscopy Centre (MC)***			
		n = 188	n = 9
	Mean (Range)	2 (1 - 7)	2 (1 - 4)

***Number of contacts with providers before MC***			
		n = 188	n = 9
	Mean (Range)	4 (1 - 12)	4 (2 - 9)

***Duration of treatment before MC (days)***			
		n = 188	n = 9
	Median (Range)	11 (1 - 161)	69 (13 - 213)*
		*Mann-Whitney Test, p = .000	

***Period of no-treatment before MC (days)***			
		n = 188	n = 9
	Median (Range)	11 (0 - 166)	194 (8 - 736)*
		*Mann-Whitney Test, p = .000	

***Total cost of help-seeking before MC (Rs.)***			
		n = 169	n = 6
	Median (Range)	330 (0 - 30735)	1375 (270 - 8500)*
		*Mann-Whitney Test, p = .014	

***Cost of help-seeking before MC as a percentage of PCI (%)***			
		n = 122	n = 4
	Median (Range)	34.21 (0 - 10245)	366.67 (80.50 -1455.06)
	Mean	176.23	567.22

Note: Percentages are calculated on valid responses.

For the outliers, help-seeking in the private medical sector adversely affected access to TB care. Twenty-year old Meenadevi was diagnosed and treated for TB for six months in the private sector. This treatment cost her Rs. 7000 (US$ 155.6), amounting to four times the monthly per capita income of Rs. 1667 (US$ 37). A month after completing the six-month course of treatment, her symptoms re-appeared. She went to a municipal hospital where she was diagnosed with TB and started on DOTS. Meenadevi's story is more or less representative of others in this group. Eight of the nine 'outliers' consulted a private practitioner (PP) before they reached the microscopy centre. For most of them (6/9), the attending PP either failed to diagnose TB or did not inform the respondents of the diagnosis. In the two cases where the attending PP did diagnose TB, respondents were not referred to the public sector for TB treatment.

In some cases, however, provider delay was influenced by respondent behaviour, particularly where respondents chose to disregard advised regimens for anti-TB treatment. Forty-year old Basappa developed a cough and was diagnosed with TB and started on anti-TB treatment by a PP about 15 days later. After two months, Basappa discontinued the treatment and went back to his native village. Five months later, his symptoms worsened and he returned to Mumbai and consulted the same PP. Following an x-ray, he was re-diagnosed with TB and advised admission to a hospital. At this point, Basappa chose to approach the municipal health care facility since he could not afford in-patient care in the private sector.

### Diagnostic delay

Diagnostic delays were on average very short (n = 216; median 4 days, range 0 to 23). Ten respondents reported diagnostic delays beyond the 95^th ^percentile (10 days). This was mainly due to factors beyond the respondents' control. For 17-year old Fatima, the illness started soon after she came to Mumbai following her marriage. After taking "*tonics and other medicines*" from a number of PPs, Fatima was advised a chest x-ray by one PP, who diagnosed TB and referred her to a municipal centre. The next day, Fatima went to the centre with her mother-in-law and gave a sputum sample. On the same day, her father took her with him to his hometown in an adjoining district. Fatima returned to the same centre a week later after her mother-in-law was informed by the health worker that her sputum sample had tested positive for TB and that she needed to complete the investigation by providing two more sputum samples in order to start treatment. Another respondent, 35-year old Kunjappa, a construction worker, left town on a contract after giving a single sputum sample. He returned to the microscopy centre eight days later when a health care provider visited his house and encouraged him to complete the investigations. Inability to produce sputum and providing saliva instead of sputum added to diagnostic delay for 19-year old Pyarelal and 25-year old Ramesh. The remaining two respondents experienced delays because of the floods that brought the city to a standstill in July 2005.

### Treatment delay

The average treatment delay was as short as the median diagnostic delay (n = 262; median 4 days, range 0 to 47 days). Thirteen respondents were beyond the 95^th ^percentile (22 days) for treatment delay. Six of the 13 respondents were diagnosed when admitted to municipal hospitals and only started on DOTS after being discharged from the hospitals. Delays after discharge from the hospital were both patient and systems-related. John, 33 years old, could not be registered for DOTS for 15 days, as the health staff at his local DOT centre was occupied with a polio eradication campaign and could not verify his home address, a pre-condition for entry into the programme. Meanwhile, John's symptoms worsened and he needed to be re-admitted to the hospital. John's TB treatment was finally started 35 days after he was diagnosed with TB.

Cultural factors also contributed to delayed initiation of treatment. Aslam, a 40-year old male who observed the religious fasts in the Islamic holy month of *Ramadaan *was diagnosed a few days after the holy month began and chose to wait until the month was over to start the treatment. Similarly, Saraswati, a 52-year old Hindu woman, went on a pilgrimage before she started the treatment.

Patients who reside in Mumbai for employment purposes tend to be without families and are dependent on co-workers and acquaintances for shelter. Frequent changes in residence within the same locality, long work hours and a reliance on these temporary 'shelters' restrict these patients' treatment support networks. For example, despite being resident in Mumbai for six years, 29-year old Dilip took nearly three weeks to find a person who could accompany him as guarantor. Twenty-two year old Pankaj was told that his treatment could start only if he had a ration card, i.e. proof of residence in the area. As he did not have one, Pankaj could only start treatment after the intervention of an NGO social worker, 45 days after he was diagnosed.

## Discussion

Against the backdrop of Mumbai's highly successful TB control programme, the study focused attention on the NSP patients who fall outside of the 95^th ^percentile for delays at each stage of treatment seeking, from onset of symptoms until initiation of DOTS. For a programme that enrols more than 30,000 patients per year [2], the three to five percent 'outliers' for each of the delay indicators amount to a sizeable number. Causes for delays among TB patients have been explored by a number of researchers in settings across the world [15-21]. However, only a few community-based studies have explored the realities of patients who fail to access treatment through the national TB control programmes [22-24]. Identifying and highlighting the cases of 'outliers', i.e., individuals who 'just made it' into the programme - as opposed to discarding them from population based statistical profiles of a TB control programme - ensures that an analysis of missed opportunities is comprehensive and grounded in local level realities [25].

The thread common to all 'outliers' was their poverty: 'outliers' had a significantly lower per capita income than the rest of the sample. The 'outliers' came from households with a median per capita income which is below the poverty line [26]. The cases presented in this paper highlight the ways in which a lack of economic and material resources is compounded with social, structural and environmental factors, as has been documented by others [27-29]. Economic poverty translates into particular social vulnerabilities that impact individual treatment-seeking pathways. The influence of these vulnerabilities differs at various stages of the help-seeking process.

Numerous studies have discussed age, gender, access to financial resources [17,18,20,21,24,30] as well as poor physical access to diagnostic facilities [19,22] as predictors of higher patient delay. However, for the majority of respondents in this study, the excellent transport and public health infrastructure in Mumbai compensated for most of these barriers. This is seen in the duration of patient delays which, on average, was shorter than that reported in other Indian studies [16,17,22] as well as elsewhere [18-21]. Rather, it is generalized, chronic illness conditions that raised the threshold for perceiving a condition of 'serious' morbidity and result in longer patient delays. The gap between perceived and clinically relevant symptoms has been well-documented in India [22,24,31] and South-East Asia [32,33]. Especially among the poor, acknowledgement of an illness as an abnormal state of being - and one that deserves care - is a function of affordability.

This study confirms the importance of the private sector as a preferred source of TB care [34,35] and the barrier it poses in accessing good quality TB treatment for some patients [16,22,36]. Provider delays were incurred as a result of PPs' failure to detect TB, to disclose the diagnosis to the respondent, and or to refer them to the public sector when diagnosed. On their own, the respondents turned to public sector facilities only when unable to afford the costs in the private sector, as documented elsewhere in India [37]. Surprisingly, awareness about availability of free TB medicines and history of utilisation of municipal health services did not encourage respondents to use public sector health services.

Treatment 'shopping' has often been cited as a reason for higher provider delay [38-40]. However, this study found that the 'outliers' for provider delay tended not to 'shop', but rather stayed with a provider for longer than the rest of the sample, and consequently incurred higher treatment costs. These individuals represent the predicament of those who can barely afford private sector treatment, yet tend to avoid the public sector that might have provided them with better quality TB care at no cost.

Once patients entered the RNTCP in Mumbai, they fared on average very well. With the exception of one case, diagnostic delay in 'outliers' was a result of circumstances beyond patients' control. Fatima's story highlights the situation of most young married women who have limited access to resources and no say in decision-making, even when it concerns their own health. Fatima's return to the microscopy centre was achieved through the programme's communication with decision makers from her family - an essential positive step by the programme. Dilip's case illustrates the particular vulnerability of urban migrants living in the city without strong social support networks. An earlier study conducted in Mumbai showed that one-fifth of the patients who did not complete the investigations for diagnosis of TB had left Mumbai to return to their native places [14]. This contrasts with the results of a study conducted across four Indian states, in which the main reasons for not completing the diagnostic procedures were systems-related [25].

Delays in initiation of treatment, however, can be equally attributed to systems- and patient-related factors. Routine administrative procedures involved in registering the patient for DOTS become obstacles for those who lack effective treatment support systems [17]. At the same time, there are a few patients who choose not to initiate treatment, even when enrolled.

Difficulties faced by the 'outliers' in accessing DOTS provide an indication of the barriers facing those who fail to reach the programme. However, our approach relies on the assumption that 'outliers' and the patients who are 'missed out' by the RNTCP share socio-demographic, economic and cultural characteristics. We used reported data from patients to describe all delay variables and hence did not exclude the period of 21 days (the 'latent' period that defines a suspect) from the reported care-seeking pathway. In the case of TB, as with other diseases that have a gradual onset, patients often find it difficult to state the exact time of onset of symptoms. Further, clinical practice in a high prevalence population, as that represented by Mumbai city, varies from the 'ideal' programmatic and policy scenario. Clinicians do not rely exclusively on the reported duration of onset of symptoms, but often suspect TB based on their experience, and relevant clinical and social characteristics of the patient. We also did not collect information on whether the patient had been in contact with a known case of TB before being diagnosed with TB. This additional information may have partially explained why some patients sought treatment within 21 days of onset of treatment, as contacts of known cases of TB are instructed by health care providers to report symptoms suggestive of TB immediately. We were unable to verify reported provider delays due to lack of documented evidence (e.g. prescriptions, case record sheets etc) of help-seeking in private clinics.

Notwithstanding these limitations, the profiles of TB 'outliers' provide important clues as to how the programme might better reach those patients who are missing from their statistics. First, there is a need to strengthen links with private sector providers and increase their skills to suspect and diagnose TB early. As strongly suggested in Meenadevi's case, PPs also need to be convinced of the benefits of referring patients to the RNTCP early; this has been shown to reduce provider delays and costs for patients [40]. On the other hand, migrant patients like Dilip, might benefit from additional support, through collaborations with community-based and non-government organisations.

Second, the community needs to be sensitised to the availability of free TB treatment in the public sector. Awareness about availability of the services has been shown elsewhere to influence the patient's choice of a health provider for TB treatment [32]. In the present study, past use of public health services did not prompt current use of the public sector. It is essential to strengthen the quality of care provided through the public sector to ensure greater satisfaction to its users.

Finally, as illustrated in the encouraging cases of Kunjappa and Fatima as well as the discouraging cases of Aslam and Saraswati, health care provider-patient communication can play an important role in shaping patients' perceptions of the illness, treatment processes and the quality of health care providers themselves. Both diagnostic and treatment delay can be addressed by patient-centred communication, specifically at the time of the first visit for sputum microscopy and at the time of disclosing the diagnosis to the patient. The programme needs to ensure that patients assign due importance to the test and comply with the instructions. As in Fatima's story, the involvement of a family member in cases of patients who do not have much say regarding their own treatment (e.g. young people, women), may help to reduce delays. There is also a need for innovative strategies to reach out to those patients who do not initiate treatment despite being enrolled in the programme because of other priorities. Counselling that takes local social and cultural context (e.g. gender dynamics, religious proscriptions) might prove useful in reducing treatment delays for patients like Aslam and Saraswati.

## Conclusion

The success of public health programmes is often measured through achievement of targets in terms of coverage, detection and cure rates. However, target-driven success rates focus, by default, on those who access the programme. Focusing on 'outliers' in a successful programme, helps us to identify the unique vulnerabilities of individuals, who respond differently to programme directives and approaches. For programme managers, a focus on 'outliers' provides a less resource- and time-intensive alternative to community-based studies for understanding the missed opportunities for enabling comprehensive coverage of the programme in poor urban settings.

## Competing interests

The authors declare that they have no competing interests.

## Authors' contributions

AP contributed to the design of the study, supervised the data collection, analysed and interpreted the data and wrote and revised successive drafts of the paper. KK contributed to the interpretation of data, drafting, revision and editing of the paper. HG, AB and JP provided critical feedback and editorial suggestions. SR conceived and designed the study, supervised data collection, interpreted the data and helped revise successive drafts of the paper. All authors (AP, KK, HG, AB, JP, SR) read and approved the final manuscript.

## Pre-publication history

The pre-publication history for this paper can be accessed here:

http://www.biomedcentral.com/1471-2458/10/263/prepub

## References

[B1] World Health Organization WHO Report 2006: Global tuberculosis control, surveillance, planning, financing Geneva: WHO/HTM/TB/2006.362

[B2] Central TB Division, Government of India TB India 2009: RNTCP status report, New Delhi http://www.tbcindia.org

[B3] Singh V Jaiswal A Porter JDH Ogden JA Sarin R Sharma PP Arora VK Jain RC TB control, poverty, and vulnerability in Delhi, India Trop Med Int Health 2002 7 8 693 700 10.1046/j.1365-3156.2002.00909.x 12167096

[B4] Jaiswal A Singh V Ogden JA Porter JDH Sharma PP Sarin R Arora VK Jain RC Adherence to tuberculosis treatment: lessons from the urban setting of Delhi, India Trop Med Int Health 2003 8 7 625 633 10.1046/j.1365-3156.2003.01061.x 12828545

[B5] Ashford LS Gwatkin DR Yazbeck AS Designing health and population programs to reach the poor 2006 Washington, D.C.: Population Reference Bureau

[B6] Government of India Census 2001 http://www.censusindia.gov.in/

[B7] Municipal Corporation of Greater Mumbai Mumbai City Development Plan 2005 - 2025 http://www.karmayog.org/docs/mumbai2005-25.pdf

[B8] Government of Maharashtra Economic survey of Maharashtra 2005-2006. Directorate of Economics and Statistics, Planning Department, Government of Maharashtra 2006 http://www.maharashtra.gov.in/english/ecoSurvey/ecoSurvey1/esmint45/ind_e.pdf

[B9] Coker R McKee M Atun R Dimitrova B Dodonova E Kuznetsov S Drobniewski F Risk factors for pulmonary tuberculosis in Russia: case-control study BMJ 2006 332 85 87 10.1136/bmj.38684.687940.80 16339219 PMC1326930

[B10] Jackson S Sleigh AC Wang GJ Liu XL Poverty and the economic effects of TB in rural China Int J Tuberc Lung Dis 2006 10 10 1104 1110 17044202

[B11] David AM Mercado SP Becker KE Mugisha F The prevention and control of HIV/AIDS, TB and vector-borne diseases in informal settlements: Challenges, opportunities and insights J Urban Health 2007 84 1 i65 i74 10.1007/s11524-007-9183-5 17431796 PMC1891654

[B12] Kemp JR Mann G Nhlema Simwaka B Salaniponi FML Squire SB Can Malawi's poor afford free tuberculosis services? Patient and household costs associated with a tuberculosis diagnosis in Lilongwe Bull World Health Organ 2007 85 580 585 10.2471/BLT.06.033167 17768515 PMC2636388

[B13] Rangan S Ambe G Borremans N Zallocco D Porter J The Mumbai experience in building field level partnerships for DOTS implementation Tuberculosis 2003 83 165 172 10.1016/S1472-9792(02)00070-7 12758207

[B14] Ambe G Lonnroth K Dholakia J Copreaux J Zignol M Borremans N Uplekar M Every provider counts: effect of a comprehensive public-private mix approach for TB control in a large metropolitan area in India Int J of Tuberc Lung Dis 2005 9 5 562 568 15875930

[B15] Rajeswari R Chandrasekaran V Suhadev M Sivasubramaniam S Sudha G Renu G Factors associated with patient and health system delays in the diagnosis of tuberculosis in South India Int J Tuberc Lung Dis 2005 6 9 789 795 12234134

[B16] Balasubramanian R Garg R Santha T Gopi PG Subramani R Chandrasekaran V Thomas A Rajeswari R Anandakrishnan S Perumal M Niruparani C Sudha G Jaggarajamma K Frieden TR Narayanan PR Gender disparities in tuberculosis: report from a rural DOTS programme in South India Int J Tuberc Lung Dis 2004 8 3 323 332 15139471

[B17] Eastwood SV Hill PC A gender-focused qualitative study of barriers to accessing tuberculosis treatment in The Gambia, West Africa Int J Tuberc Lung Dis 2004 8 1 70 75 14974748

[B18] Cambanis A Yassin MA Ramsay A Squire SB Abride I Cuevas LE Rural poverty and delayed presentation to tuberculosis services in Ethiopia Trop Med Int Health 2005 10 4 330 335 10.1111/j.1365-3156.2005.01393.x 15807796

[B19] Chiang CY Chang CT Chang RE Li CT Huang RM Patient and health system delays in the diagnosis and treatment of tuberculosis in Southern Taiwan Int J Tuberc Lung Dis 2005 9 9 1006 1012 16158893

[B20] Karim F Islam MA Chowdhury AMR Johansson E Diwan VK Gender differences in delays in diagnosis and treatment of tuberculosis Health Policy and Planning 2007 22 329 334 10.1093/heapol/czm026 17698889

[B21] Tobgay KJ Sarma PS Thankappan KR Predictors of treatment delays for tuberculosis in Sikkim Natl Med J India 2006 19 2 60 63 16756190

[B22] Buu TN Lonnroth K Quy HT Initial defaulting in the National Tuberculosis Programme in Ho Chi Minh City, Vietnam: a survey of extent, reasons and alternative actions taken following default Int J Tuberc Lung Dis 2003 7 8 735 741 12921149

[B23] Santha T Garg R Frieden TR Subramani R Gopi PG Chandrasekaran V Selvakumar N Thomas A Rajeswari R Balasubramanian R Kolappan C Narayanan PR Are community surveys to detect tuberculosis in high prevalence areas useful? Results of a comparative study from Tiruvallur District, South India Int J Tuberc Lung Dis 2003 7 3 258 265 12661841

[B24] Dandona R Dandona L Mishra A Dhingra S Venkatagopalakrishna K Chauhan LS Utilization of and barriers to public sector tuberculosis services in India The Natl Med J India 2004 17 6 292 299 15736548

[B25] Porter JDH Epidemiological reflections of the contribution of anthropology to public health policy and practice J Biosoc Sci 2006 38 133 144 10.1017/S0021932005001070 16283952

[B26] Guruswamy M Abraham RJ Defining hunger and malnutrition: The poverty line is a starvation line 2006 http://www.infochangeindia.org/agenda6_04print.jsp

[B27] Salaniponi FML Harries AD Banda HT Kang'ombe C Mphasa N Mwale A Upindi B Nyirenda TE Banerjee A Boeree MJ Care seeking behaviour and diagnostic processes in patients with smear-positive pulmonary tuberculosis in Malawi Int J Tuberc Lung Dis 2000 4 4 327 332 10777081

[B28] MacKian S A review of health seeking behaviour: problems and prospects. DfID Health Systems Development Programme, Working Paper HSD/WP/0503 2001 University of Manchester

[B29] Lienhardt C Ogden JA Tuberculosis control in resource-poor countries: have we reached the limits of the universal paradigm? Trop Med Int Health 2004 9 7 833 841 10.1111/j.1365-3156.2004.01273.x 15228495

[B30] Lonnroth K Thuong LM Linh PD Diwan VK Delay and discontinuity-a survey of TB patients' search of a diagnosis in a diversified health care system Int J Tuberc Lung Dis 1999 3 11 992 1000 10587321

[B31] Kielmann K Bentley M Thresholds of perceived morbidity among women in a peri-urban community of Maharashtra, India: Conceptual and methodological issues Journal of Health Psychology 2003 8 525 538 10.1177/13591053030085005 19177715

[B32] Auer C Sarol J Jr Tanner M Weiss M Health seeking and perceived causes of tuberculosis among patients in Manila, Philippines Trop Med Int Health 2000 5 9 648 656 10.1046/j.1365-3156.2000.00615.x 11044280

[B33] Lonnroth K Tran TU Thuong LM Quy HT Diwan V Can I afford free treatment?: Perceived consequences of health care provider choices among people with tuberculosis in Ho Chi Minh City, Vietnam Soc Sci Med 2001 52 935 948 10.1016/S0277-9536(00)00195-7 11234866

[B34] Arora VK Sarin R Lonnroth K Feasibility and effectiveness of a public-private mix project for improved TB control in Delhi, India Int J Tuberc Lung Dis 2003 7 12 1131 1138 14677887

[B35] Arora VK Gupta R Public-Private Mix: A prioritisation under RNTCP - An Indian perspective Indian J Chest Dis Allied Sci 2004 46 27 37 14870866

[B36] Rangan S The Public-Private Mix in India's Revised National Tuberculosis Control Programme - An Update J Indian Med Assoc 2003 101 3 161 163 14603964

[B37] Sudha G Nirupa C Rajasakthivel M Sivasubramanian S Sundaram V Bhatt S Subramaniam K Thiruvalluvan E Mathew R Renu G Santha T Factors influencing the care-seeking behaviour of chest symptomatics : a communitybased study involving rural and urban population in Tamil Nadu, South India Trop Med Int Health 2003 8 4 336 341 10.1046/j.1365-3156.2003.01010.x 12667153

[B38] Needham DM Foster SD Tomlinson G Godfrey-Faussett P Socio-economic, gender and health services factors affecting diagnostic delay for tuberculosis patients in urban Zambia Tro Med Int Health 2001 6 4 256 259 10.1046/j.1365-3156.2001.00709.x 11348515

[B39] Kiwuwa MS Charles K Harriet MK Patient and health service delay in pulmonary tuberculosis patients attending a referral hospital: a cross-sectional study BMC Public Health 2005 5 122 10.1186/1471-2458-5-122 16307685 PMC1310609

[B40] Kelkar-Khambete A Kielmann K Pawar S Porter J Inamdar V Datye A Rangan S India's Revised National Tuberculosis Control Programme: looking beyond detection and cure Int J Tuberc Lung Dis 2008 12 1 1 6 18173883

